# RATEmiRs: the rat atlas of tissue-specific and enriched miRNAs database

**DOI:** 10.1186/s12864-018-5220-x

**Published:** 2018-11-19

**Authors:** Pierre R. Bushel, Florian Caiment, Han Wu, Raegan O’Lone, Frank Day, John Calley, Aaron Smith, Jianying Li

**Affiliations:** 1 Biostatistics and Computational Biology Branch, Research Triangle Park, NC USA; 2 Microarray and Genome Informatics Group, National Institute of Environmental Health Sciences, P.O. Box 12233, Research Triangle Park, NC 27709 USA; 3 Department of Toxicogenomics, Maastricht University, Maastricht, The Netherlands; 4 Department of Discovery and Development Statistics, Lilly Research Laboratories, Lilly Corporate Center, Indianapolis, Indiana, USA; 5 Health and Environmental Sciences Institute, Washington, D.C., USA; 6 Office of Scientific Computing, National Institute of Environmental Health Sciences,, Reaserch Triangle Park, NC USA; 7 Department of TTX Bioinformatics, Lilly Research Laboratories, Lilly Corporate Center, Indianapolis, Indiana, USA; 8 Department of Investigative Toxicology, Non-Clinical Safety Assessment and Pathology, Lilly Research Laboratories, Lilly Corporate Center, Indianapolis, Indiana, USA; 9 Integrative Bioinformatics, National Institute of Environmental Health Sciences,, Research Triangle Park, NC USA; 10 Kelly Government Solutions,, Research Triangle Park, NC USA

**Keywords:** miRNA, RNA-Seq, Database, Tissue-enriched, Tissue-specific, Organ-specific

## Abstract

**Background:**

MicroRNAs (miRNAs) regulate gene expression and have been targeted as indicators of environmental/toxicologic stressors. Using the data from our deep sequencing of miRNAs in an extensive sampling of rat tissues, we developed a database called RATEmiRs for the Rat Atlas of Tissue-specific and Enriched miRNAs to allow users to dynamically determine mature-, iso- and pre-miR expression abundance, enrichment and specificity in rat tissues and organs.

**Results:**

Illumina sequencing count data from mapped reads and meta data from the miRNA body atlas consisting of 21 and 23 tissues (14 organs) of toxicologic interest from 12 to 13 week old male and female Sprague Dawley rats respectively, were managed in a relational database with a user-friendly query interface. Data-driven pipelines are available to tailor the identification of tissue-enriched (TE) and tissue-specific (TS) miRNAs. Data-driven organ-specific (OS) pipelines reveal miRNAs that are expressed predominately in a given organ. A user-driven approach is also available to assess the tissue expression of user-specified miRNAs. Using one tissue vs other tissues and tissue(s) of an organ vs other organs, we illustrate the utility of RATEmiRs to facilitate the identification of candidate miRNAs. As a use case example, RATEmiRs revealed two TS miRNAs in the liver: rno-miR-122-3p and rno-miR-122-5p. When liver is compared to just the brain tissues for example, rno-miR-192-5p, rno-miR-193-3p, rno-miR-203b-3p, rno-miR-3559-5p, rno-miR-802-3p and rno-miR-802-5p are also detected as abundantly expressed in liver. As another example, 55 miRNAs from the RATEmiRs query of ileum vs brain tissues overlapped with miRNAs identified from the same comparison of tissues in an independent, publicly available dataset of 10 week old male rat microarray data suggesting that these miRNAs are likely not age-specific, platform-specific nor pipeline-dependent. Lastly, we identified 10 miRNAs that have conserved tissue/organ-specific expression between the rat and human species.

**Conclusions:**

RATEmiRs provides a new platform for identification of TE, TS and OS miRNAs in a broad array of rat tissues. RATEmiRs is available at: https://www.niehs.nih.gov/ratemirs

## Background

MicroRNAs (miRNAs), short non-coding RNA molecules of approximately 22 nucleotides in length, regulate gene expression by binding to the 3′ untranslated regions of messenger RNAs (mRNAs) to inhibit translation or directly causing degradation of the transcripts [1–4]. miRNAs have recently become of great interest as molecular targets for disease intervention and as tissue-specific biofluid based biomarkers [5, 6]. For example, measuring miR-122 in the blood, the expression of which is specific to the liver, has been investigated as a potential biomarker for various types of liver disease or dysfunction [7–9]. Having the ability to accurately detect the level of expression of miRNAs in tissues and organs is key to exploiting their full potential as modern day therapeutic targets.

Several recent efforts surveyed the expression of miRNAs in the tissues and organs of humans, mice, rats and other species [10–14]. miRNA sequences have been shown to be highly conserved between certain species [15]. Except for the beagle dog miRNA tissue atlas, currently the databases of miRNA expression are largely based on microarray analysis and/or offer limited analytics supporting tissue-enrichment (TE) and tissue-specificity (TS). RNA-sequencing (RNA-Seq) has recently out performed microarray in the analysis and utilization of gene expression in clinical and regulatory settings [16, 17]. Smith et al. [18] used three different bioinformatics pipeline analyses of deep miRNA-Seq data to survey the baseline expression of miRNAs in 21 and 23 tissues of male and female Sprague Dawley rats respectively, that make up 14 different organs. This rat miRNAs body atlas data is publicly available but currently, there is no useful way of performing meta-analysis of the data across tissues and within organs.

We developed a database called RATEmiRs for the **R**at **A**tlas of **T**issue-specific and **E**nriched **miRNAs** to allow users to determine mature-, iso- and pre-miR expression abundance, enrichment and specificity in Sprague Dawley rat tissues and organs. Using the RNA-Seq data from the rat miRNA body atlas, we developed user-friendly query interfaces to dynamically detect TE, TS and organ-specific (OS) miRNAs across three different bioinformatics pipelines (Data-Driven) based on 1) non-negative matrix factorization (NMF) [19] by Eli Lilly, 2) quasi-Poisson modeling by the National Institute of Environmental Health Sciences (NIEHS) and 3) percentage of total mapped reads by Maastricht University. In addition, a User-Driven interface is available to query the tissue expression of user-specified miRNAs. Functionality is implemented in RATEmiRs to compare abundantly expressed miRNAs from two or all three of the pipelines, to plot and display the expression of the data and to download results. Using one tissue vs other tissues and tissues of an organ vs other organs, we illustrate the utility of RATEmiRs to facilitate the identification of abundantly expressed miRNAs.

### Construction and content

**Fig. 1:**
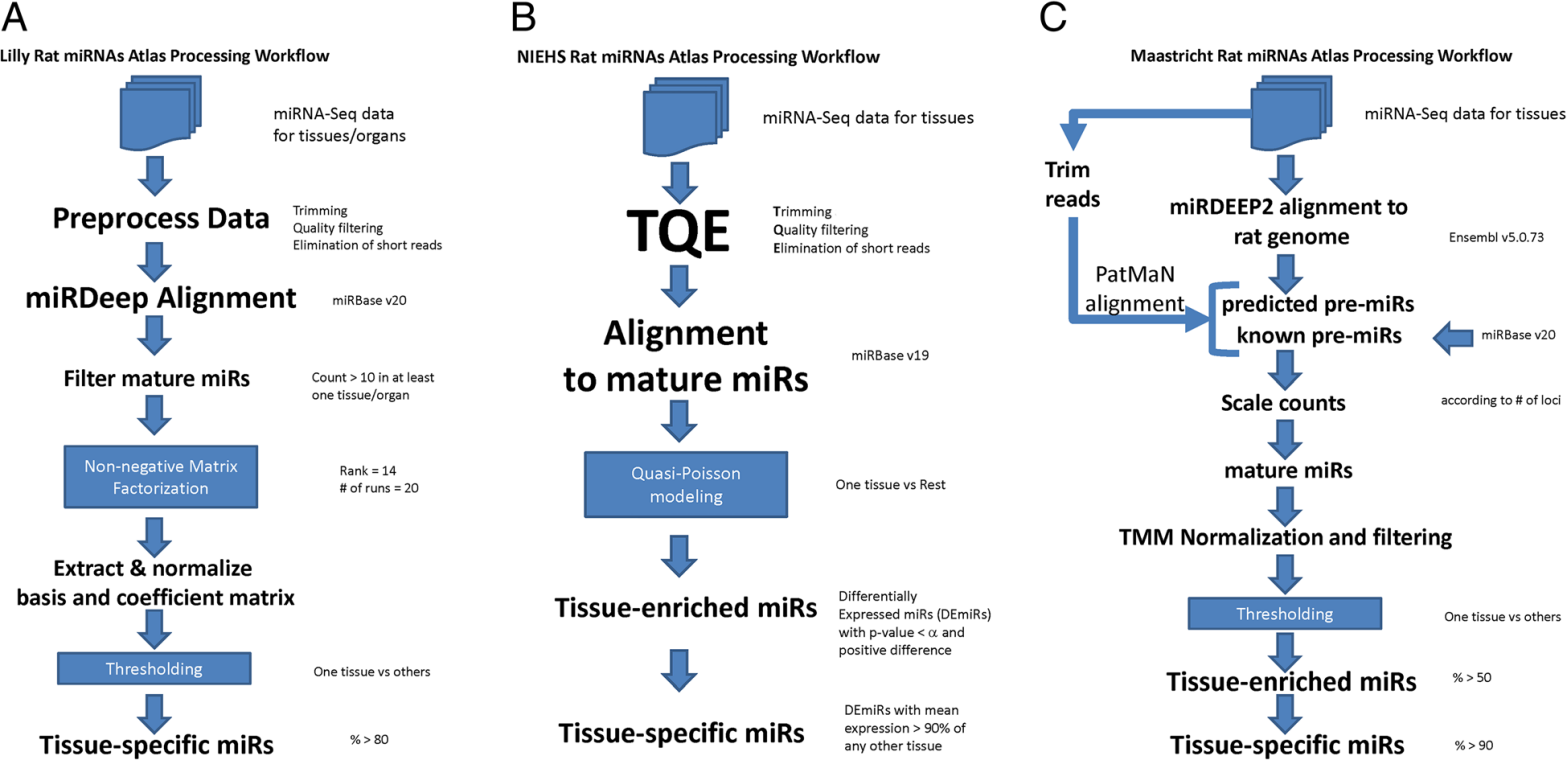
RATEmiRs pipelines. A) Eli Lilly, B) NIEHS and C) Maastricht University. Each pipeline has a workflow which preprocesses the data, aligns the reads and performs an analysis to detect TE, TS and OS miRNAs

Tissues (Table 1) from the organs of five male and five female Sprague Dawley rats 12–13 weeks in age were harvested, preserved and total RNA extracted. Detailed information on the tissues collected and miRNA sequence libraries are as previously described [18]. Illumina sequencing of the miRNAs extracted from the 215 tissue samples was performed by Illumina HiSeq 2000 analysis generating 50 bp single-end reads with 4–5 million reads per sample. The raw data is made available within the Gene Expression Omnibus (GEO) [20, 21] through GEO Series accession number GSE78031. Three separate bioinformatics pipelines (Fig. 1) processed the data as previously described [18]. Table 2 provides a comparison of the analysis steps for each pipeline. The strengths and limitations of the core analysis methods implemented into each pipeline are shown in Table 3. Below is a detailed description of each pipeline.

**Table 1:** Sample sizes of each tissue for each pipeline

	Pipeline sample sizes		
Tissues	Lilly	NIEHS	Maastricht
Adrenal	10	10	10
Muscle biceps^e^	10	10	10
Brainstem^d^	10	10	10
Cerebellum^d^	10	10	10
Cerebrum^d^	10	10	10
Cortex^a^	10	10	10
Dorsal root ganglion (DGR/Uk)	10	4	10
Duodenum^c^	10	9	10
Stomach glandular (Gln)^b^	10	10	10
Heart	10	10	10
Hippocampus^d^	10	10	10
Ileum^c^	10	8	10
Jejunum^c^	10	10	10
Kidney^a^	10	10	10
Liver	10	10	10
Medulla^a^	10	10	10
Stomach non-glandular (NGln)^b^	10	10	10
Ovary	5	5	5
Pancreas	10	10	10
Muscle soleus^e^	10	10	10
Testicle	5	1	5
Uterus	5	5	5
Whole Blood	10	6	10

Denotation of tissues that comprise of an organ

^a^Kidney; ^b^Stomach; ^c^Intestine; ^d^Brain; ^e^Muscle

**Table 2:** Comparison of pipelines analysis steps

Analysis steps	Eli Lilly	NIEHS	Maastricht University
Preprocessing	Trim adapters	Trim adapters	Trim adapters
	Quality filtering	Quality filtering	
	Elimination of short reads	Elimination of short reads	Elimination of short reads
Alignment	miRDeep using miRBase v20	BWA using miRBase v19	Pre-alignment: miRDeep2 using Ensembl v5.0.73
			Post-Alignment: PatMaN using predicted miRs and miRBase v20
Read count filtering	Yes (i.e. > 10 in at least one tissue/organ)	Yes (i.e. > 3 in at least one tissue/organ)	NA
Scaling	NA	NA	According to # of loci
Pre-Normalization	NA	TPM^a^	TMM
Statistical method	Non-negative Matrix Factorization	Quasi-Poisson modeling	Percentage of total mapped read counts
Comparison	One vs All	One vs Rest	One vs All
Post-Normalization	Of basis (W) and coefficient (H) matrices	NA	NA
Tissue-enriched thresholding	% of total expression (i.e. 60% in more than one tissue/organ)	*p*-value (i.e. < 0.05)	% of total expression (i.e. > 50%)
Tissue/organ-specific thresholding	% of total expression (i.e. > 80% in one tissue/organ)	*p*-value and % of expression (i.e. <0.05 and > 90%)	% of total expression (i.e. > 90%)
Data for display	TMM	TMM	TMM

^a^Prior to statistical analysis, a transformation from floats to integers was performed by ceiling the data

**Table 3:** Strengths and limitations of core analysis methods implemented into the pipelines

Core Analysis Methods Implemented	Strengths	Limitations
NMF	Factors are interpretable	Factorization (W,H matrices) is not always unique
	Reduces dimensions of the data	No statistical inference
	Fast computation	Convergence can be slow
Quasi-Poisson	Has underlying statistical inference	Model dependency and complexity
	Computational simplicity	No probability distribution or log-likelihood
	Accounts for over-dispersion of the data	Supported by asymptotic (large sample) theory in special cases
		Requires normalization and transformation of the data
% Total Mapped Reads	Proportion basis offers an intuitive relationship to relative expression	Requires normalization of the data
	Easy to implement	No statistical inference

### Eli Lilly – Non-negative matrix factorization

FastQ files were preprocessed to remove adapter sequences, filtered to discard reads < 17 bp in length and trimmed. Trimmed reads containing an ‘N’ were discarded. Identical sequences from the same sample were combined into a single sequence. Using miRDeep2 [22], reads were aligned to known miRNAs from rat miRBase v20 [23–27]. Each isomiR (variant of a mature miRNA) sequence in an alignment was associated with the corresponding mature miRNA identifier. A read is identified as <miR> − pre if it was found to map to a miRNA precursor but not with the mature miRNA sequence that is expected. If a given sequence was identified as mapping to two or more precursors, it was associated with all potential names. Sequences that did not align were compared to known miRNAs from other species (mouse, human then *C. elegans*).

Tissue level counts (summing over all the animals for a tissue) were aggregated to the organ level by selecting the maximum read count of the tissues for a given organ. Other choices of aggregation included using the average, which might dilute the organ level signal. As we are equally interested in miRNAs that are expressed at all levels, we used a technique called NMF [19] to find TE/TS/OS miRNAs for each level of expression. To determine the expression level of each miRNA, a two-component mixture of Poisson distributions was fitted to the tissue counts data. The larger component of the two-component Poisson mixture model relates to high expression miRNA, whereas the smaller component corresponds to the low miRNA expression. This is to ensure that NMF is applied to miRNAs that have similar levels of expression. Otherwise, we are likely to miss miRNAs that are expressed at medium or low level. Let X be the organ level count matrix of dimension N by M, where N is the number of miRNAs and M is the 14 organs. NMF was used to decompose the non-negative matrix X into 2 non-negative matrices W and H. Each column of W explains a miRNA factor group and each column of H defines the expression of the miRNA factor group corresponding to the particular organ type. Based on this decomposition, OS miRNAs are identified by their high expression level for the organ. This method was also applied to detect TE and TS miRNAs. We impose the constraint that a TS miRNA has to be OS.

### NIEHS – Quasi-Poisson statistical modeling

FastQ files with the RNA-Seq reads were checked for quality and preprocessed using recursive **t**rimming of the adapters, quality filtering at Q ≤ 20 and **e**limination (TQE) of reads < 14 or > 25 bp long. Using the Burrows-Wheeler Alignment (BWA) tool [28], reads passing the TQE filtering were aligned to rat miRBase v19 [23–27]. Read counts from perfect matches were summarized for each mature miRNA. Seventeen of the 215 samples had too few reads remaining after TQE and alignment and were therefore removed from analysis.

To detect TE miRNAs, a one-vs-rest strategy was adopted. Abundantly expressed miRNAs in one tissue vs all other tissues were identified using a quasi-Poisson (Quasi-Seq) model [29]. Significantly expressed miRNAs as TE were detected at a nominal *p*-value < α and with a positive difference. To detect TS miRNAs, a percentile criteria was used to select any TE miRNA which had a mean expression > a number of percentage points above the maximum mean expression from any of the other tissues. OS miRNAs were identified with the same model and manner for detecting TE miRNAs except that an organ-vs-rest quasi-Poisson modeling was implemented.

### Maastricht – Percentage of total mapped reads

Using miRDeep2 [22] FastQ files were mapped to the rat genome (version 5.0.73 from Ensembl [30]). To retain predicted miRNA precursors with a score of 1 or above, we parsed the output then trimmed raw reads and discarded any with a size < 16 or > 35 bp. Using PatMaN, a fast short read mapping software [31], we mapped trimmed reads to rat precursor miRNAs or generated de novo from the miRDeep2 prediction. To generate pre-normalized count data, we parsed the PatMaN output in order to assign a unique name to each unique sequence and then divided the total read count of each by the number of assigned loci for the miRNA. Finally, the data was normalized by the trimmed mean of M-values method (TMM) [32] and then filtered to remove all miRNAs where the TMM was < 10 in all of the samples.

We defined a miRNA as TE or TS/OS when the proportion of reads aligned for a single tissue (or organ) was greater than 0.5 or 0.9 of the total reads aligned respectively. To identify isomiRs, we first converted the raw count number of each given isomiR to the proportion of expression compared to the mature miRNA. Then by comparing the proportions, we report the miRNAs for which the most expressed isomiRs differs between all of the tissues.

### Database implementation

**Fig. 2:**
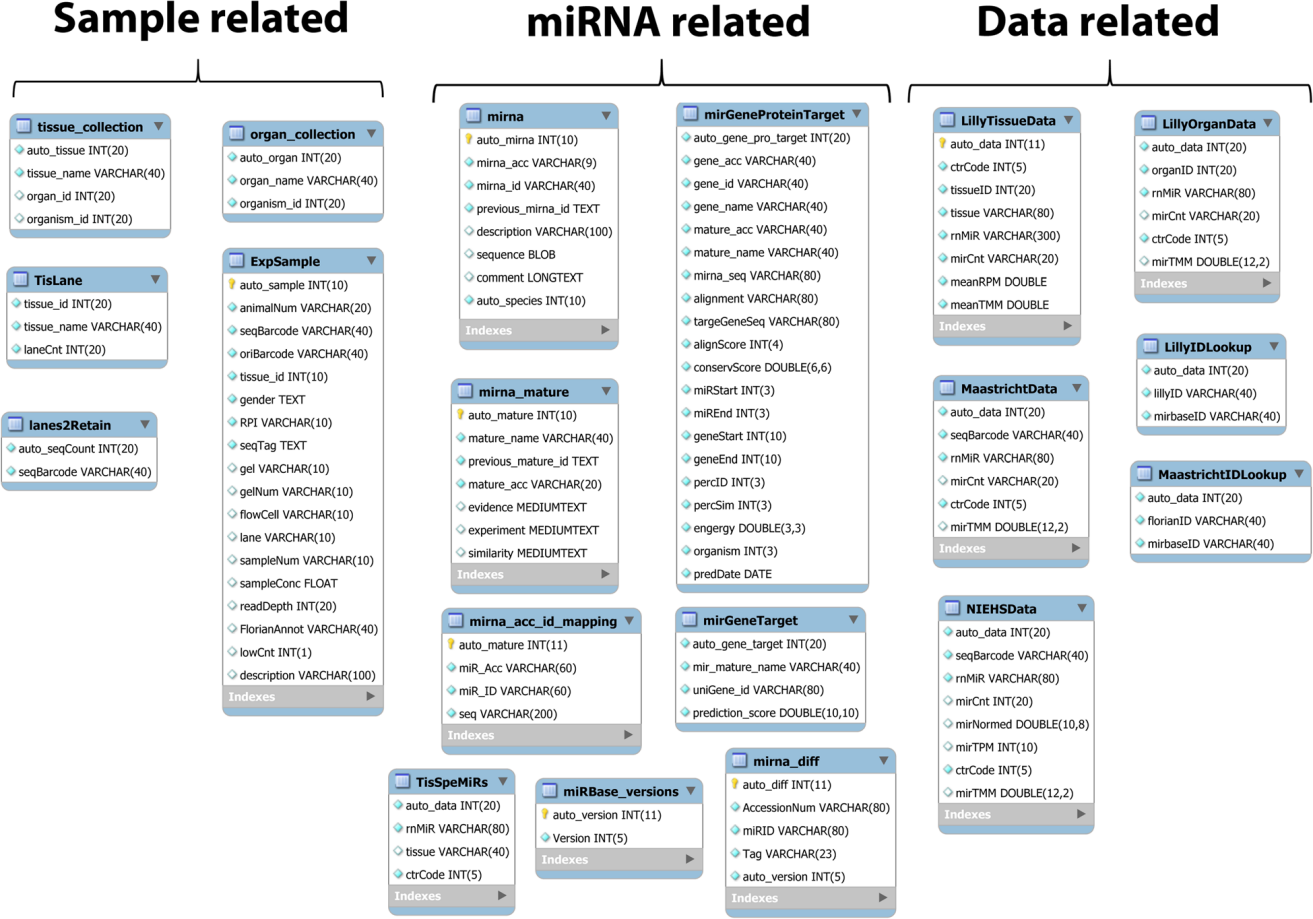
RATEmiRs database tables. Core RATEmiRs database tables organized by their utility: sample-related, miRNA-related and data-related. The mirna-diff table tracks the versioning of miRBase so that the annotation of the miRNA IDs are updated dynamically

**Fig. 3:**
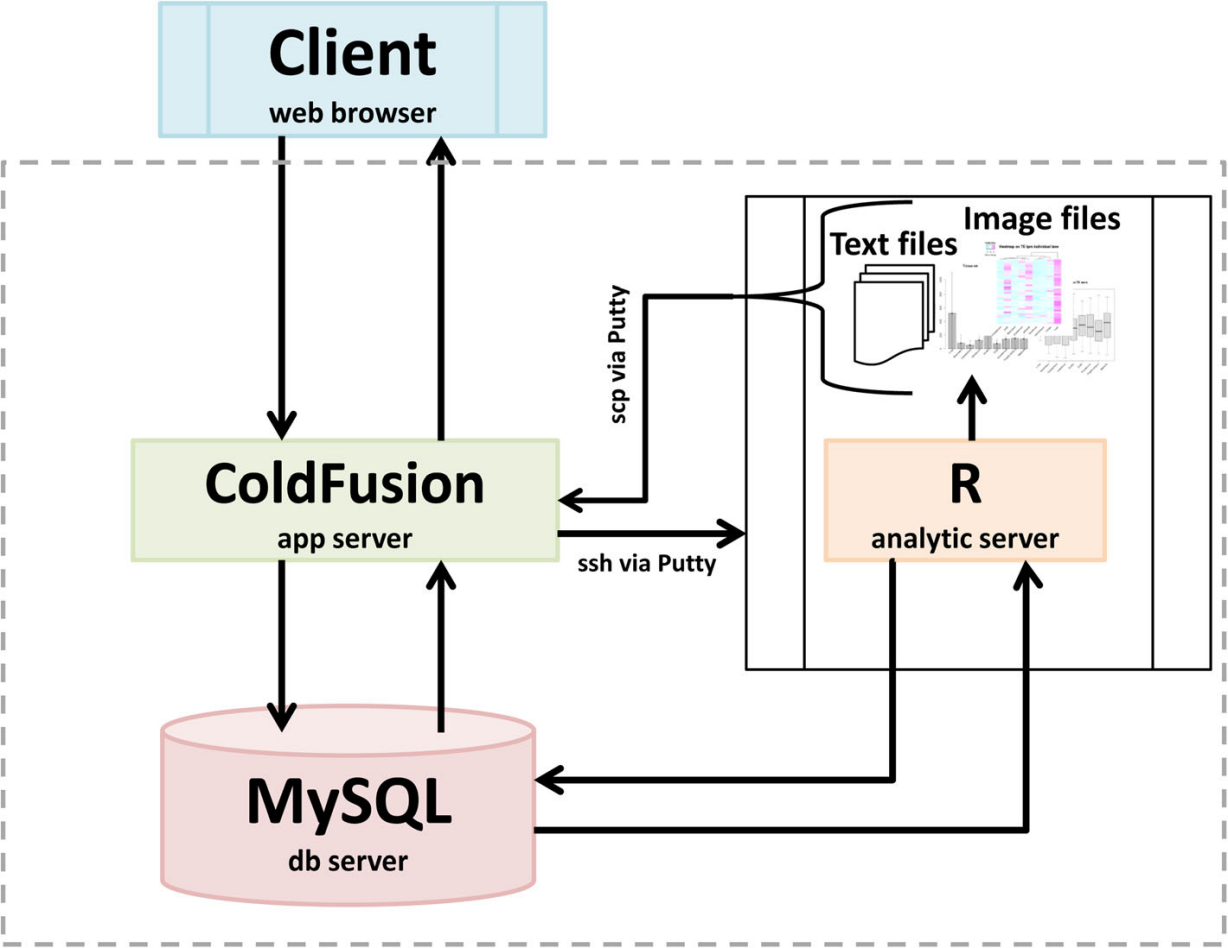
RATEmiRs database web application schema. The components of the RATEmiRs web application database are shown. The client web browsers currently supported are Chrome and Firefox. Requests from the web browser are received by the ColdFusion web application server which in turns executes R scripts. The MySQL database stores the data and the R analytic server queries the database to perform the analyses on the data and generate the output files. Data files are transferred using a secured shell (ssh) file transfer and copy protocol (scp). The ColdFusion server also queries the database and provides the web browser client the results in web page tables and embedded graphics as well as downloadable text and image files

The RATEmiRs database contains tables (Fig. 2) to store data related to the samples, miRNAs and specific analysis pipeline. Eli Lilly’s mature-, pre- and isomiRs read count and normalized data, NIEHS’ mature miRNAs read count and normalized data and Maastricht’s mature-, pre- and isomiRs count and normalized data were loaded into a MySQL database [33]. A previous claim of TMM poor performance for miRNA sequencing data [34] has been disproved and attributed to an error in utilization of the normalization method [35]. Rigorous optimization of miRNA sequencing data revealed that TMM is recommended for count normalization [36, 37]. The count data from all three pipelines were normalized by TMM in order to harmonize the transcript measurements for display purposes. Tissues are grouped (flagged in the TisLane table) according to the organ they were extracted from. As shown in Table 1, 14 tissues are denoted as derived from the kidney, stomach, intestine, brain or muscle and along with the other nine tissues, make up a total of 14 organs. miRBase and miRDB [38] external resources are used to update the annotation of the miRNAs according to the current version and provide the structure of the database respectively. Individual pipelines have their respective naming conventions of the miRNAs. However, when the query for abundant miRNAs compares two or all three pipelines, the annotation of the miRNAs are reconciled by lookup tables and then presented in the Venn diagram overlap with a common miRBase identifier. A ColdFusion® web application server manages the user web requests to query the database (Fig. 3). R scripts [39] for each pipeline process the data by way of an analytical server.

## Utility and discussion

**Fig. 4:**
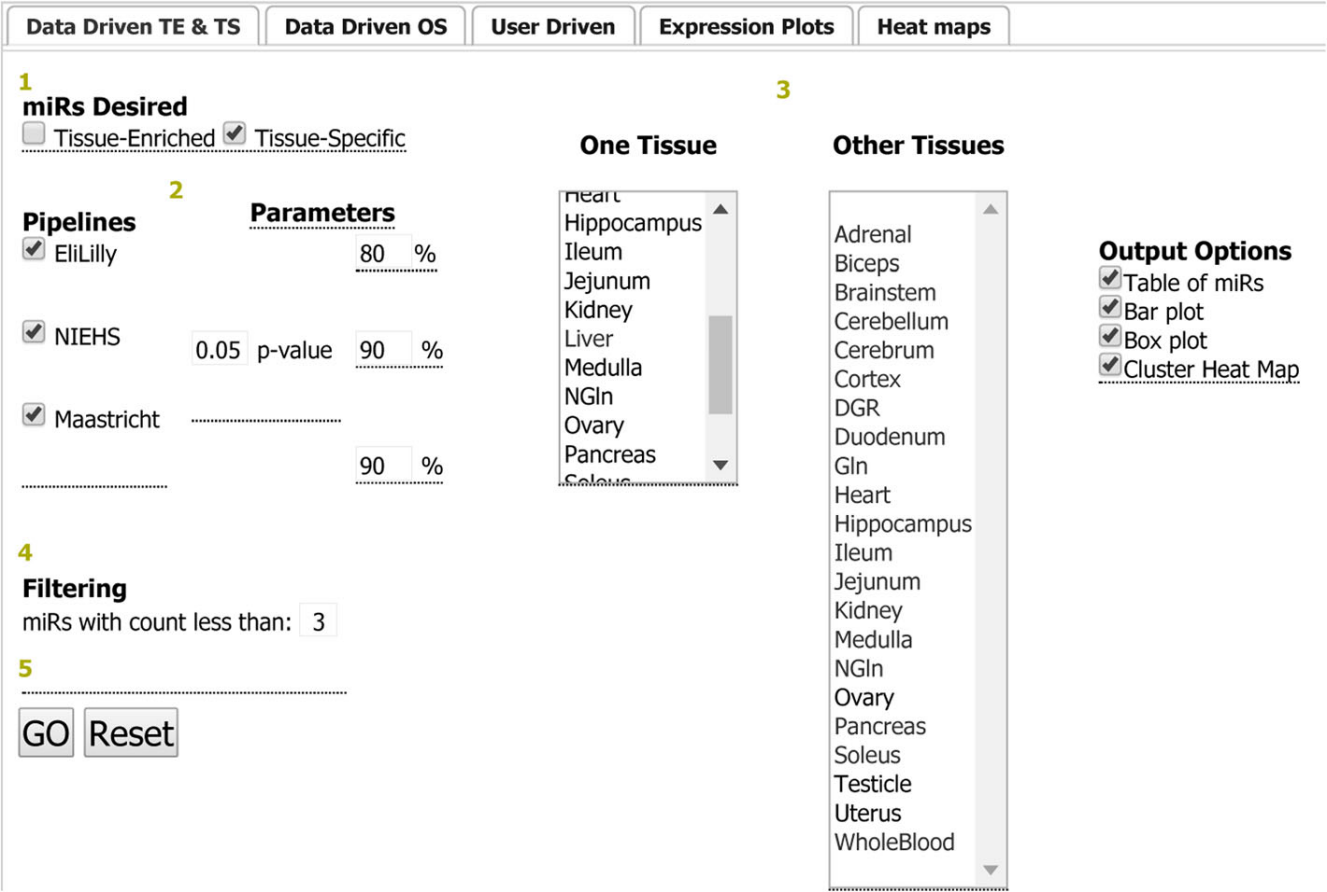
RATEmiRs interface. The Data Driven TE and TS analysis tab is shown. Other tabs are for Data Driven OS, User Driven, viewing expression plots and viewing heat maps. The steps to perform an analysis are denoted by the numbered circles. Mouseover of a bolded title or parameter setting describes the function or parameter setting value used in the analysis. The query output is presented in the tab that launched the analysis. The profile plots of the miRNAs’ expression and the heat maps of the miRNAs’ expression are presented in the Expression Plots tab and the Heat maps tab respectively

There are two ways of querying the data through the RATEmiRs interface (Fig. 4). The Data-Driven approach computationally identifies TE, TS or OS miRNAs depending on the pipeline(s) chosen and the parameter(s) selected. Users can select one tissue to compare to two or more other tissues or an organ represented by or one or more tissues. The User Driven entrez permits a user to enter in miRBase IDs in order to display the expression determined by selected pipelines. The query interfaces have no more than 5 steps (numbered in circles) to follow to perform an analysis for identification of TE, TS, OS or user-defined miRNAs. For identifying TE or TS miRNAs:

1. Select which type is desired
2. Select the analysis pipeline(s) and adjust parameters if needed
3. Select one tissue to compare with two or more selected other tissues
4. If necessary, adjust the filtering of miRNAs by read counts
5. Hit Go

For identification of OS miRNAs:

1. Select the analysis pipeline(s) and adjust parameters if needed
2. Select one organ
3. If necessary, adjust the filtering of miRNAs by read counts
4. Hit Go

To obtain the expression of user-defined miRNAs:

1. Select the analysis pipeline(s)
2. Enter a list of miRNAs (one per line) using mature miRBase identifiers
3. Select two or more tissues
4. Hit Go

**Fig. 5:**
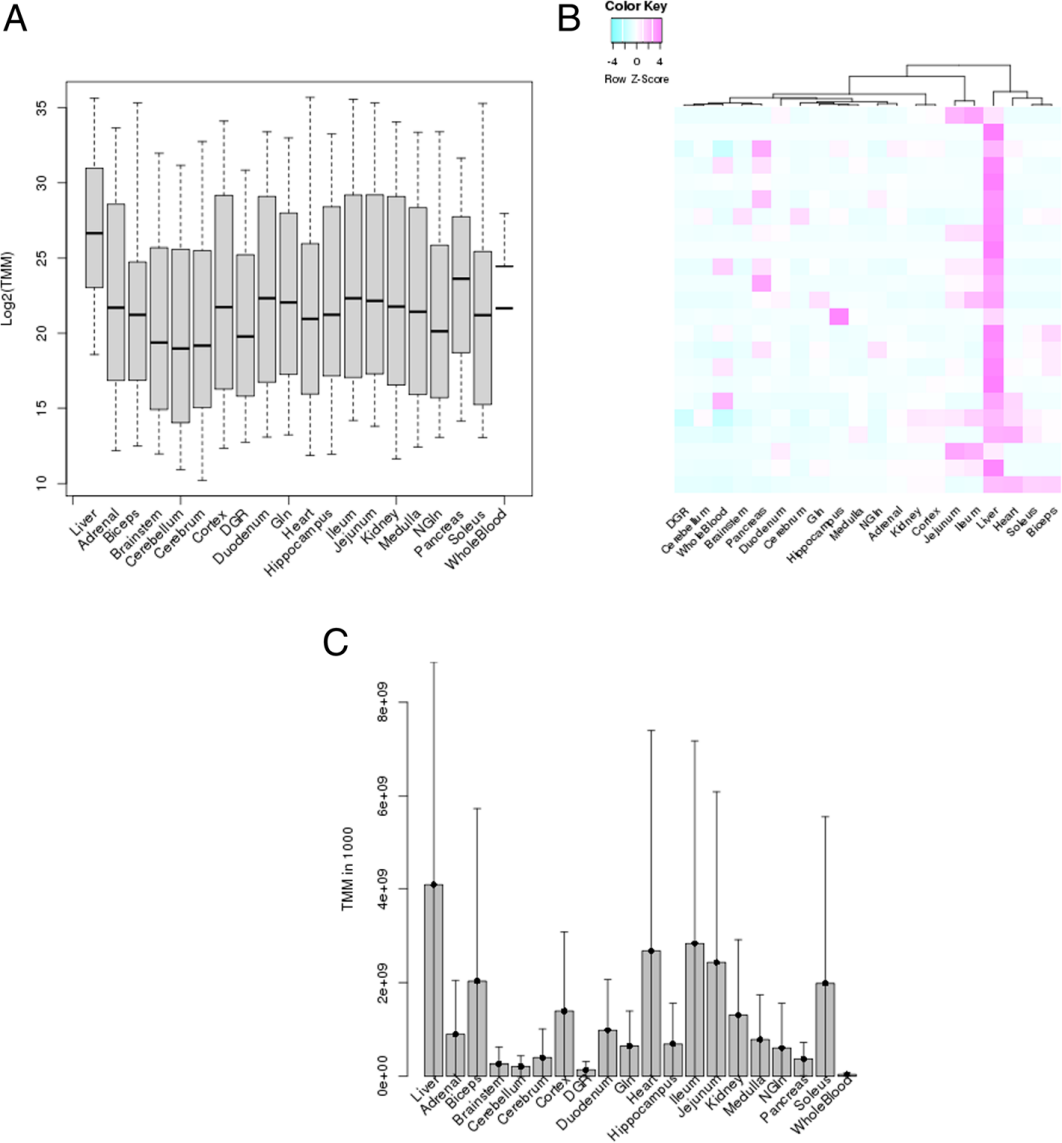
Example of NIEHS pipeline TE query analysis results from liver vs other tissues (except the ovary, testicle and uterus). A) Box plot of the average expression of TE miRNAs within a tissue displaying the distribution of data based on the minimum, first quartile, median, third quartile, and maximum values. The x-axis is the tissues/organs and the y-axis is the log base 2 of TMM. B) Cluster analysis of the average expression of TE miRNAs (rows) in the tissues (columns). The clustering is performed in R using the heatmap.2 function with default parameters (dist for the distance metric and hclust to perform the hierarchical clustering) and scaling/centering of the data by row (miRNAs). The heat map colors are represented by z-scores as depicted in the legend. C) Bar chart of the average expression of TE miRNAs with the vertical bar representing the standard deviation of the mean. The x-axis is tissues/organs and the y-axis is TMM

**Fig. 6:**
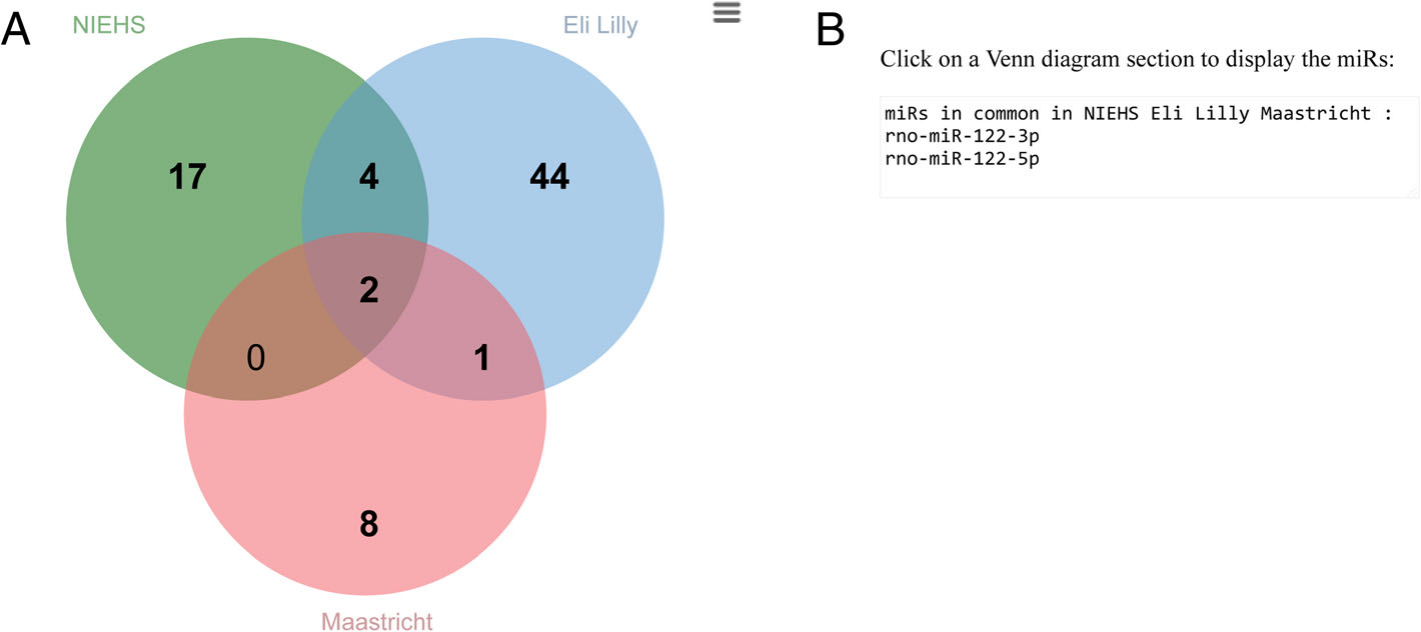
Overlap of TE miRNAs. A) Venn diagram and B) list showing intersection of liver TE miRNAs from the Eli Lilly, NIEHS and Maastricht University pipelines in a liver vs other tissues (except the ovary, testicle and uterus) comparison. Clicking on a number in the Venn diagram (A) will display the overlapping or unique miRNAs in the display box (B). The image of the Venn diagram (PNG or SVG) and the miRNAs (unique and intersections) as lists in a file (CSV) are downloadable by clicking the menu (three lines in the upper right-hand corner of the Venn diagram)

Query and analysis results are presented in the form of tables with the mean expression (averaged across the male and female biological replicates) within each tissue or organ (Table 4) or as shown in Fig. 5, bar plots of the distribution of the miRNAs within each tissue or organ, box plots illustrating the spread of the miRNAs in each tissue and a cluster analysis representing the similarity of the expression of the miRNAs across the samples. Finally, if more than one pipeline is selected, a Venn diagram is produced depicting the overlap of the miRNAs detected as TE, TS or OS (Fig. 6). Overlapping miRNAs suggest higher confidence in them as, abundant, enriched or specific in a tissue or organ whereas nonoverlapping ones may represent pipeline-specific analysis results or miRNAs not detected as abundant by the other pipelines. All the result sets are downloadable.

**Table 4:** Abundance of the miRNAs in the liver vs brain tissues

miRNA ID	Liver	Brainstem	Cerebellum	Cerebrum	Hippocampus
rno-miR-101b-3p	9673.302	259.239	333.532	239.783	325.597
rno-miR-122-3p	4009.98	0.168	0.045	0.134	0.092
rno-miR-122-5p	69,427.419	0.428	0.87	0.69	1.574
rno-miR-142-3p	363.858	26.372	10.863	12.454	61.031
rno-miR-142-5p	3637.916	118.302	71.591	102.668	682.414
rno-miR-144-3p	579.159	22.386	16.137	11.014	16.39
rno-miR-144-5p	679.886	37.424	22.018	18.614	20.869
rno-miR-192-5p	468,832.18	2273.309	4705.168	1628.248	49,459.205
rno-miR-193-3p	1737.21	15.646	5.535	10.126	71.69
rno-miR-194-5p	29,022.858	222.803	1005.578	154.443	4888.132
rno-miR-203b-3p	346.805	0.549	0.34	1.926	5.414
rno-miR-21-5p	159,245.015	3854.307	1345.632	1652.192	15,147.209
rno-miR-22-3p	1,236,523.448	96,603.646	55,374.293	166,371.249	235,026.152
rno-miR-22-5p	444.407	63.992	28.289	87.822	84.76
rno-miR-31a-3p	135.821	4.969	0.734	4.788	12.909
rno-miR-31a-5p	2931.165	168.328	32.897	158.017	583.204
rno-miR-339-5p	314.445	49.849	25.719	27.668	43.617
rno-miR-3559-5p	550.276	11.454	11.879	13.587	34.687
rno-miR-365-3p	470.536	26.121	10.247	22.187	60.292
rno-miR-378a-3p	16,034.731	383.201	604.102	379.106	1496.621
rno-miR-378a-5p	412.681	12.512	54.307	8.676	41.054
rno-miR-451-5p	1297.679	81.875	47.434	43.356	53.144
rno-miR-6329	158.695	15.135	17.172	11.838	23.729
rno-miR-802-3p	2405.619	0.794	0.317	2.248	50.689
rno-miR-802-5p	294.88	0.093	0	0.027	6.983
rno-miR-92a-1-5p	270.871	10.879	2.949	8.154	37.219
rno-miR-92a-3p	16,055.859	197.271	58.193	158.198	198.4

Expression represented as TMM. Based on the NIEHS pipeline with p-value < 0.01, miRNA expression ≥80 percentage points above the maximum mean expression within any of the other brain organ tissues and miRNA mean expression in liver > 100 TMM

**Fig. 7:**
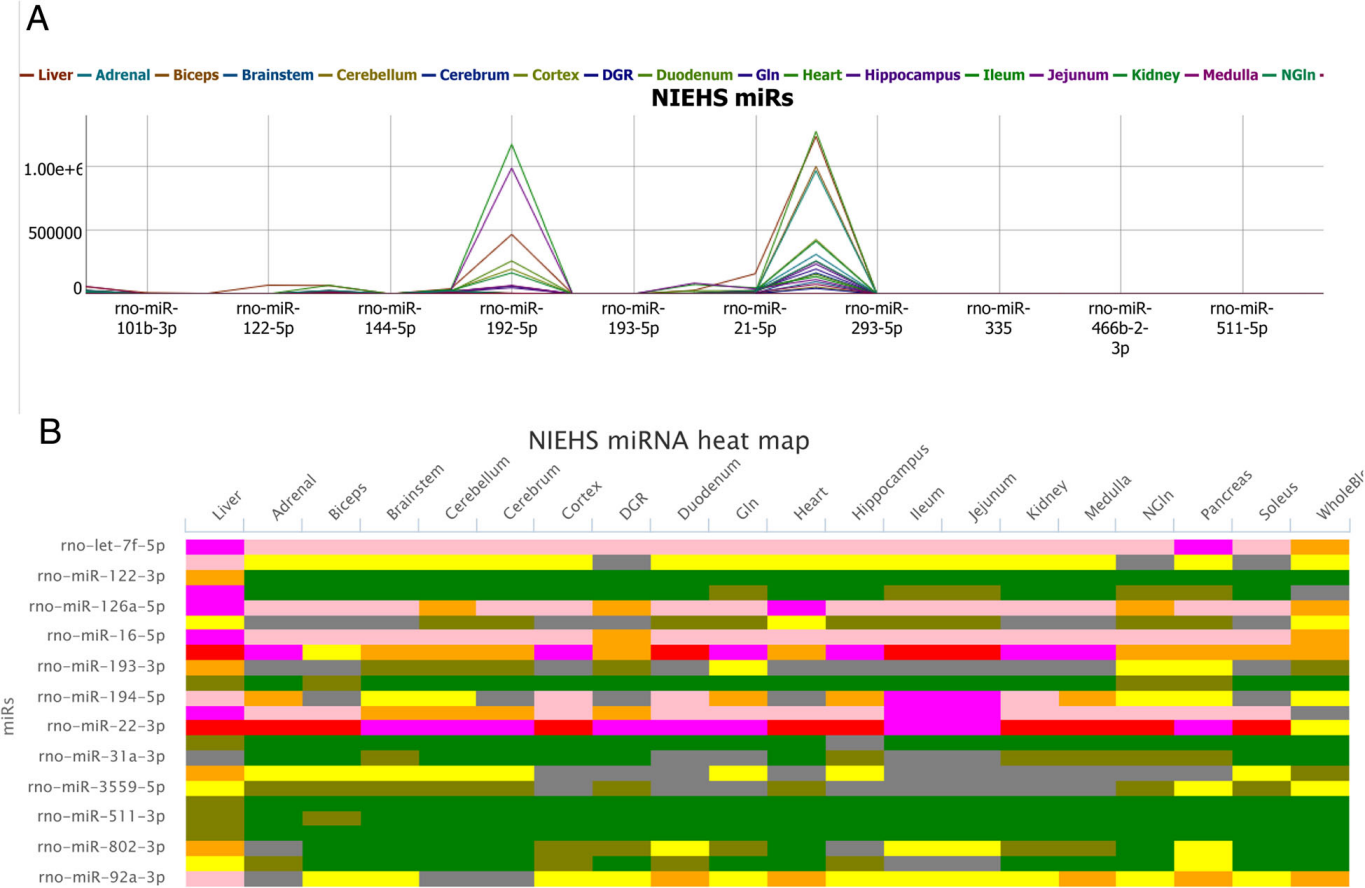
Expression plot and heatmap of TE miRNAs from the NIEHS pipeline in a liver vs other tissues (except the ovary, testicle and uterus). A) Expression profile plot with the miRNAs in the x-axis and TMM in the y-axis. Each miRNA is color coded and when the profiles are mouseovered, the TMM value for each miRNA is displayed. B) Heat map of the miRNA expression. The x-axis is the tissues/organs and the y-axis is the miRNAs. The color for the heat map expression is the TMM data scaled to values between − 4 and + 4. Mouseover of a cell in the heat map displays the miRNA, its scaled expression and the tissue/organ of expression. The image (PNG, JPEG, PDF and SVG formats) of the heat map is downloadable by clicking the menu (three lines in the upper right-hand corner of the heat map)

The Expression Figures tabs contains a dynamically generated expression plot (Fig. 7a) which can be zoomed in to reveal the levels of expression of the miRNAs and a heat map (Fig. 7b) that displays the expression of the miRNAs as a color representation according to the data scaled between − 4 and + 4. The heat map can be downloaded as an image file.

The data included in the RATEmiRs database is from male and female Sprague Dawley rats with ages ranging between 12 and 13 weeks. To assess whether age or platform affects the miRNAs identified as TE, we compared the miRNAs overlapping the three pipelines in ileum vs brain tissues (cerebellum, cerebrum and hippocampus) against those obtained in the same comparison of miRNAs obtained from 10 week old male Sprague Dawley rats where the samples were assayed on customized Agilent miRNA microarrays (GEO accession number GSE52754) [13]. The RATEmiRs TE querying parameters were set as follows:

Eli Lilly: threshold = 40%.

NIEHS: *p*-value = 0.05.

Maastricht: threshold = 50%.

There were 55 miRNAs identified in the overlap of the three RATEmiRs pipelines (Table 5). The GEO2R empirical Bayes statistics [40, 41] analysis (limma with log2 FC > 0.5 and FDR <  0.05) of the 10 weeks in age male rat data was based on ileum vs brain tissues (cerebellum and cerebrum [cortex, hippocampus and thalamus]) and yielded 456 miRNAs. All 55 miRNAs from the RATEmiRs query of the 12–13 week old male and female rat data were identified by the GEO2R query of the 10 week old male rat data suggesting that these miRNAs are not age-specific, platform-specific nor pipeline-dependent.

**Table 5:** TE miRNAs that overlap the three RATEmiRs pipelines in ileum vs brain tissues

miRNA IDs	
rno-miR-1-3p	rno-miR-200a-3p
rno-miR-10a-3p	rno-miR-200a-5p
rno-miR-10a-5p	rno-miR-200b-3p
rno-miR-130b-3p	rno-miR-200b-5p
rno-miR-130b-5p	rno-miR-200c-3p
rno-miR-133a-3p	rno-miR-203a-3p
rno-miR-133a-5p	rno-miR-203b-3p
rno-miR-141-3p	rno-miR-20a-5p
rno-miR-141-5p	rno-miR-20b-3p
rno-miR-142-3p	rno-miR-20b-5p
rno-miR-142-5p	rno-miR-21-3p
rno-miR-143-3p	rno-miR-21-5p
rno-miR-143-5p	rno-miR-223-3p
rno-miR-145-3p	rno-miR-27a-5p
rno-miR-145-5p	rno-miR-28-3p
rno-miR-146a-3p	rno-miR-301b-3p
rno-miR-146a-5p	rno-miR-31a-3p
rno-miR-15b-3p	rno-miR-31a-5p
rno-miR-15b-5p	rno-miR-3558-5p
rno-miR-183-3p	rno-miR-3559-3p
rno-miR-183-5p	rno-miR-3559-5p
rno-miR-18a-5p	rno-miR-363-3p
rno-miR-192-3p	rno-miR-375-3p
rno-miR-192-5p	rno-miR-802-3p
rno-miR-196c-3p	rno-miR-802-5p
rno-miR-196c-5p	rno-miR-92a-1-5p
rno-miR-19a-3p	rno-miR-96-5p
rno-miR-19a-5p	

Conservation of tissue/organ-specificity between rat and human was observed for 10 miRNAs (Table 6). Using the default parameters for each of the three pipelines in RATEmiRs, TS or OS miRNAs in the rat liver, heart, pancreas, intestine or testis tissues/organs also exhibited tissue-specificity in those same tissues in the human as revealed by the Human Tissue miRNA Atlas [10]. The tissue-specificity index defined as

**Table 6:** Tissue/organ-specific miRNAs conserved between rat and human

Tissue/Organ	Expression Type	miRNA	Lilly Rat TSI	NIEHS Rat TSI	Maastricht Rat TSI	Human TSI (body 1/body 2)
Liver	TS	rno-miR-122-3p	1.00	0.97	0.97	1/0.91
Liver	TS	rno-miR-122-5p	1.00	0.97	0.97	0.99/0.94
Heart	OS	rno-miR-208a-3p	1.00	0.98	0.96	0.98/0.96
Pancreas	TS	rno-miR-216a-5p	1.00	0.97	0.96	0.9/0.92
Pancreas	TS	rno-miR-216b-5p	0.99	0.96	0.96	0.72/0.95
Pancreas	TS	rno-miR-217-3p	0.99	0.96	0.96	0.92/0.96
Pancreas	TS	rno-miR-217-5p	0.99	0.97	0.96	0.92/0.96
Intestine^a^	OS	rno-miR-215-5p	0.94	–	0.94	–
Testis^b^	TS	rno-miR-509-3p	0.99	0.95	0.99	0.96/0.96
Testis^b^	TS	rno-miR-509-5p	0.99	0.95	0.98	0.98/0.98
Several	Ubiquitous	rno-miR-21-3p	0.57	0.71	0.71	0.8/0.69

*TS* tissue-specific, *TSI* Tissue Specificity Index, *OS* organ-specific. Specificity determined by RATEmiRs analysis in a one vs all other tissues and using the default parameters for each

Shown are the rat miRNAs that have specific expression and overlap with specific expression of human miRNA from the Human Tissue Atlas

^a^Indicates that the miRNA was not an annotated feature in the NIEHS pipeline

^b^Denotes that the NIEHS pipeline did not detect the miRNAs as specific

RATEmiRs TSI computed from TMM data and the human TSI computed from the Human Tissue Atlas quantile normalized data

$$ {\mathrm{TSI}}_j=\frac{\sum_{i=1}^N\left(1-{x}_{ij}\right)}{N-1} $$

where *x*_*ij*_ is the TMM expression of miRNA *j* in tissue *i* normalized by the maximal expression of miRNA *j* in any of the *N* tissues [10, 42]. TSI ranges between 0 and 1 with measures closer to 0 indicative of a miRNA expressed in many tissues and measures closer to 1 indicative of a miRNA expressed more exclusively. As shown in Table 6, the rat and human conserved TS/OS miRNAs have similar TSI measures with values > 0.9. Human hsa-miR-21 has been shown to be ubiquitously expressed and upregulated in various cancers [43]. The TSI measure for rno-miR-21-3p is ≤0.71 in the RATEmiRs rat miRNA sequencing data but is as high as 0.8 in the Human Tissue miRNA Atlas or as low as 0.69. Two human miRNAs (hsa-miR-3960 and hsa-miR-6089) were the only miRNAs ubiquitously expressed with TSI measures ≤0.32. These two aforementioned miRNAs are not represented in the rat database.

**Fig. 8:**
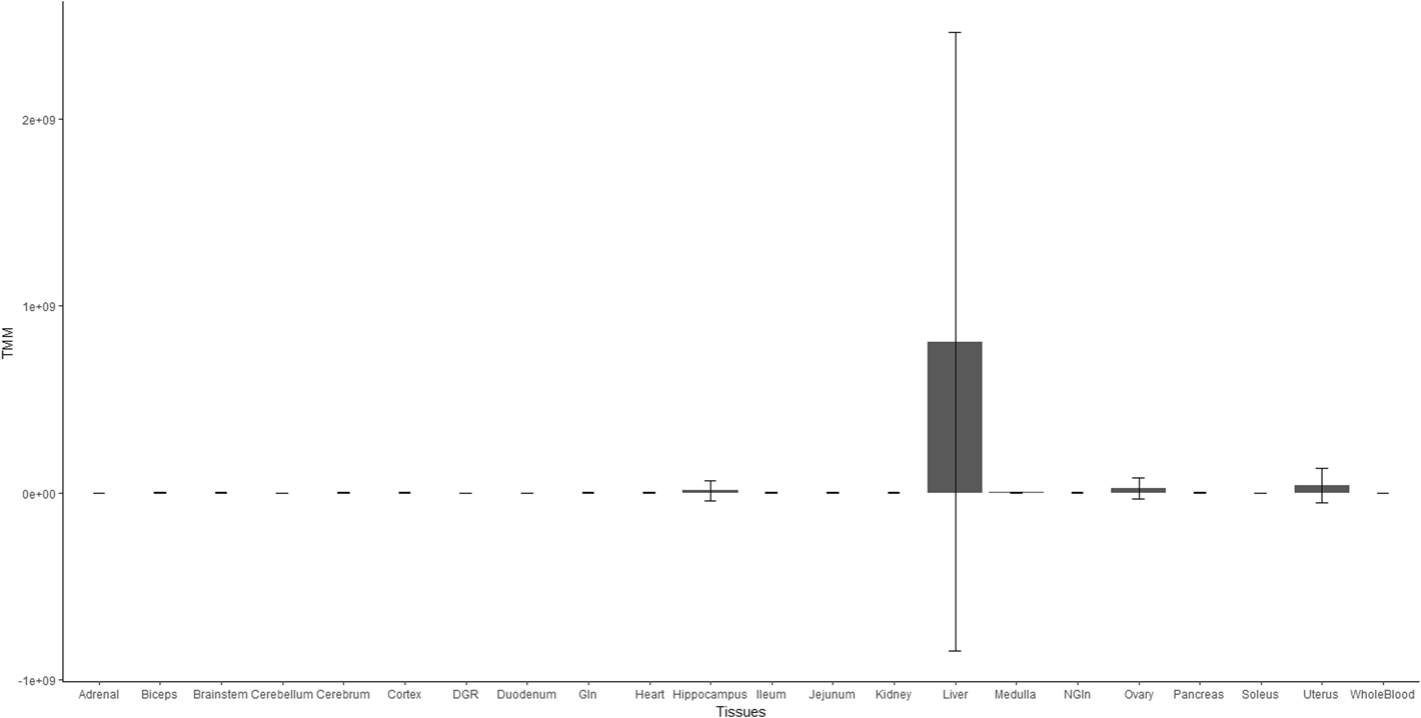
Liver-specific expression of 5 miRNAs. The Maastricht University TMM expression of 5 miRNA known to be liver-specific are plotted (rno-miR-802-5p, rno-miR-101b-3p, rno-miR-122-5p, rno-miR-192-5p and rno-miR-31-3p). The x-axis is the tissues/organs and the y-axis is TMM. The error bars represent the standard deviation of the mean expression of the 5 miRNAs

As a User Driven case scenario, 5 rat miRNAs (rno-miR-802-5p, rno-miR-101b-3p, rno-miR-122-5p, rno-miR-192-5p and rno-miR-31-3p) known to be TS in the liver [44] were queried in RATEmiRs using the User Driven entrez. Shown in Fig. 8 is a bar chart of the mean TMM expression of the 5 miRNAs from the Maastricht University pipeline in each tissue with error bars representing the standard deviations of the means. As can be seen, the miRNAs are for the most part, exclusively expressed in the liver.

The RATEmiRs tool can reliably be used to discover and query TS, TE, OS miRNAs, but the actual quantity of the miRNA may be inaccurate. T4 RNA ligases are used to ligate adapters to the miRNAs upstream of the polymerase chain reaction (PCR) and sequencing. The use of ligases may result in inaccurate quantitation of miRNAs due to inconsistent ligation efficiency caused by differences in secondary structures of miRNAs and adapters during the ligation process [45]. While ligase bias may result in inaccurate quantitation, the efficiency of ligation should be equal for a particular miRNA despite the tissue/sample of origin. This hypothesis is supported by the fact that many previously discovered TS, TE and OS miRNAs were found in the rat miRNA body atlas sequencing data [18]. However, additional methods should be employed to provide more accurate quantitation of miRNAs particularly if a miRNA is to be used as a blood-based marker of organ injury since the quantity of miRNA within a tissue may affect its utility [46]. Ligase bias in the rat miRNA body atlas data used in RATEmiRs was addressed in Smith et al. [18].

Plans for future versions of the database includes the possibility of incorporation of body tissue RNA-Seq reads of miRNAs from treated samples and implementation of additional analysis pipelines. In addition, future versions of the database will likely include partitioning of the data and analysis functionalities to identify sex differences related to the expression of the miRNAs in particular tissues and organs.

## Conclusions

The RATEmiRs database was developed to provide a user-friendly interface to the publicly available rat miRNA body map dataset. On-the-fly analysis of the expression of miRNAs in 23 tissues of Sprague Dawley rats using three different analysis pipelines is available for tissue vs tissues or organ vs organs comparisons. Comparing across multiple pipelines, tissues and organs gives the user immense analysis power and confidence in the TE, TS and OS miRNAs that overlap. Having the flexibility to narrow down miRNAs to ones which are largely expressed in a tissue or organ, or querying by user-defined miRNAs, is of valuable to scientists who want to target particular miRNAs because of their tissue specificity, comparative expression, expression abundance or biological importance. The RATEmiRs database is a useful resource not only for scientists studying miRNA biology in the rat, but also for those who are interested in some form of comparative genomics.

### Availability and requirements

Database name: RATEmiRs.

Database homepage: https://www.niehs.nih.gov/ratemirs

Browser requirement: JavaScript enabled; Chrome and Firefox web browsers are supported and recommended.

For questions regarding the RATEmiRs database functionality, contact Pierre R. Bushel (bushel@niehs.nih.gov), Jianying Li (jianying.li@nih.gov) or the RATEmiRs development team at ratemirsdevteam@niehs.nih.gov.

## Abbreviations

BWA: Burrows-Wheeler Alignment tool
GEO: Gene Expression Omnibus
HESI: Health and Environmental Sciences Institute
miRNA: MicroRNA
mRNA: messenger RNA
NIEHS: National Institute of Environmental Health Sciences
NMF: Non-negative matrix factorization
OS: Organ-specific
PCR: Polymerase Chain Reaction
RATEmiRs: Rat Atlas of Tissue-specific and Enriched miRNAs
RNA-Seq: RNA-Sequencing
TE: Tissue-enriched
TMM: Trimmed Mean of M-values
TQE: Trimming, Quality and Elimination
TS: Tissue-specific
TSI: Tissue Specificity Index
